# Blind spots of quantitative RNA-seq: the limits for assessing abundance, differential expression, and isoform switching

**DOI:** 10.1186/1471-2105-14-370

**Published:** 2013-12-24

**Authors:** Hubert Rehrauer, Lennart Opitz, Ge Tan, Lina Sieverling, Ralph Schlapbach

**Affiliations:** 1 Functional Genomics Center Zurich, University of Zurich/ETH, Zurich, Switzerland; 2 Clinical Sciences Centre, London W12 0NN, United Kingdom

## Abstract

**Background:**

RNA-seq is now widely used to quantitatively assess gene expression, expression differences and isoform switching, and promises to deliver results for the entire transcriptome. However, whether the transcriptional state of a gene can be captured accurately depends critically on library preparation, read alignment, expression estimation and the tests for differential expression and isoform switching. There are comparisons available for the individual steps but there is not yet a systematic investigation which specific genes are impacted by biases throughout the entire analysis workflow. It is especially unclear whether for a given gene, with current methods and protocols, expression changes and isoform switches can be detected.

**Results:**

For the human genes, we report their detectability under various conditions using different approaches. Overall, we find that the input material has the biggest influence and may, depending on the protocol and RNA degradation, exhibit already strong length-dependent over- and underrepresentation of transcripts. The alignment step aligns for 50% of the isoforms up to 99% of the reads correctly; only in the presence of transcript modifications mainly short isoforms will have a low alignment rate. In our dataset, we found that, depending on the aligner and the input material used, the expression estimation of up to 93% of the genes being accurate within a factor of two; with the deviations being due to ambiguous alignments. Detection of differential expression using a negative-binomial count model works reliably for our simulated data but is dependent on the count accuracy. Interestingly, using the fold-change instead of the p-value as a score for differential expression yields the same performance in the situation of three replicates and the true change being two-fold. Isoform switching is harder to detect and for at least 109 genes the isoform differences evade detection independent of the method used.

**Conclusions:**

RNA-seq is a reliable tool but the repetitive nature of the human genome makes the origin of the reads ambiguous and limits the detectability for certain genes. RNA-seq does not equally well represent isoforms independent of their size which may range from ~200nt to ~100′000nt. Researchers are advised to verify that their target genes do not have extreme properties with respect to repeated regions, GC content, and isoform length and complexity.

## Background

RNA-seq has by now in most places replaced microarrays for the analysis of the human transcriptome. Initially, it has been praised as a method that measures expression in an unbiased fashion, free of background-signal and does not require sophisticated preprocessing [1]. Without the dependency on specifically designed microarray probes, there is the expectation that in a given RNA-seq experiment the complete transcriptional state of the biological input can be captured. With the only limitation given by the sequencing depth, where for a given sequencing depth, low abundance transcripts may not be represented in the final set of reads.

The common data analysis workflows for RNA-seq consist of the steps listed in Table 1. The input material is sequenced and in the subsequent data processing the reads are aligned, the expression is estimated, differential expression is assessed and isoform switching is detected. There is a wide collection of tools available for each of these steps (for a list see e.g. [2-4]).

**Table 1:** Workflow steps towards quantitative RNA-seq together with example applications

**Input material**	**Unbiased random transcript fragments (hom. coverage)**	**Coverage bias (inhom. coverage)**	**Variable transcript start + poly-A (mod. TSS + polyA)**
**Alignment**	Global; Transcriptome + Genome [tophat]	Local; Transcriptome + Genome [STAR]	Global; Transcriptome only [RSEM]
**Abundance**	Read count (include multi-reads) [GenomicRanges: countOverlaps]	Read count(ignore multi-reads [HTSeq]	Isoform abundance model (resolve multi-reads [RSEM]
**Differential expression**	Significance using a negative binomial count model [edgeR:exactTest]	Log-ratio effect size	
**Isoform switching**	Differential isoform fractions [cuffdiff]	Differential splicing modules [DiffSplice]	Differential exons [DEXSeq]

We analyze the impact of the different types of input material and the subsequent data analysis steps on the results of quantitative RNA-seq. For each step we investigate approaches that we consider as representative for a given analysis strategy.

We investigate here to which extend these tools live up to the promise of providing accurate quantitative results. To this end, we report overall performances as well as per gene performances. We report the relative impact of library representation biases and the subsequent analysis steps on the final quantitative result, in terms of expression, expression differences and isoform switching. We tackle the question whether these results are comprehensive or whether they are limited in principle due to sequencing errors and sequence ambiguity which can not be overcome within the limits of the current technological constraints. Special attention goes here to the sequence ambiguity, which is due to sequence homology in the genomes of many species.

We perform our analysis on human RNA-seq data and in each step we apply tools that can be considered as representatives of a major analysis paradigm. The choice of the methods is subjective and does not imply superior performance compared to competing methods. For a detailed comparison of competing methods we refer to the comparison papers mentioned below.

For the alignment of RNA-seq reads there are now many different aligners available and Fonseca et al. [4] provide an overview and characterize the aligners according to their features. The two major paradigms for RNA-seq reads are

• Alignment to transcriptome: As a representative of this approach we consider RSEM [5]

• pliced alignment to genome making use of transcript annotation. Here we consider

○ tophat [6]: aligns to transcriptome sequence database and genome; tophat considers only full-length untrimmed alignments

○ STAR [7]: aligns to genome sequence complemented by a database of splice junction sequences; STAR considers also local alignments

Where the first is limited to known isoforms, and the latter has the capability to discover new isoforms.

We classify expression estimation approaches into

• Overlap counting: Count the reads that overlap a given genomic feature and directly use this as a quantitative estimate of the expression level of the feature; examples are the htseq-count [8] and the countOverlaps method in the Bioconductor package GenomicRanges [9,10]

• Isoform abundance: Model the read generation from the isoforms and estimate the isoform abundance based on the observed reads. The RSEM expression estimation follows this approach.

The overlap counting approach has the advantage that it can generate a gene level expression estimate without the need for knowing which specific isoforms are expressed. On the downside they are bound to confuse isoform switching with differential expression [11]. Further, in the absence of a read generating model (see e.g. [12], they cannot make use of additional information for resolving ambiguously aligned reads to the same degree as for example RSEM does. Ambiguity occurs if a read aligns to multiple genomic positions or if for a given genomic position multiple overlapping features are defined. The count methods deal with ambiguity by either assigning the read to all compatible features (multiple counts), discarding the read (no count), assigning a fractional count that is reciprocal to the multiplicity. If overlapping isoforms at a given gene locus are prevalent throughout the genome, as it is true for the human genome, the counting approaches can only deliver reasonable expression estimates at the gene locus level, not at the isoform level. The isoform abundance approaches explicitly deal with these multi-reads within a statistical model; a comprehensive overview of the models is given by Pachter *et al.*[12].

For differential expression, the use of negative binomial models that reflect the counting nature of the expression estimates is widely in use and an extensive performance comparison is available [13]. We apply as a representative the edgeR [14] package and additionally use the simple log-ratio as an effect-size estimator for the expression change.

For the investigation whether there is a change in the relative abundances of the different isoforms that may be generated from a given gene there is again a variety of methods. In our comparison we do not discriminate between the different molecular mechanisms like alternative splicing, alternative transcription start sites, etc. that may be the cause of an observed isoform switch. Existing approaches either look at entire isoforms, like cuffdiff2 [11], or at specific expression events, like DEXSeq (exon usage) [15] and DiffSplice (junction and exon usage) [16]; a very good overview is given in Alamancos *et al.*[3]. Again the approaches range from making full use of the annotated isoforms to complete de novo detection of splicing events simply based on local read and junction coverage.

With the methods being available and in use, it is unclear how accurate an entire workflow actually is. While Fonseca *et al.*[4] report the number of reads aligned for each aligner, they do not assess which genes are affected. Soneson *et al.*[13] compare the performance of the hypothesis test for differential expression under the assumption that the counts may have noise due to biological and technical variation. But they do not consider any systematic bias that might affect the counts of specific genes caused by wrong read alignments. In contrast to such horizontal comparison papers, this paper reports the performance of entire data analysis workflows and provides how well individual genes and isoforms can be quantified and how well differences can be assessed.

## Results and discussion

Our analysis discusses the options listed in Table 1. In order to measure the accuracy of RNA-seq, we use simulated data generated with the Flux Simulator [17] where we can control the 5′-to-3′ bias of the transcript coverage, variations in transcription starts and variable poly-A tails. We use all RefSeq isoforms present in the UCSC hg19 genome annotation and simulate the data such that each isoform has same baseline transcript abundance. This is different from the biological situation but ensures that we obtain reads from every annotated isoform. For every sample we generate 10 Mio single-end reads of length 101 bp using the default error profile that the Flux Simulator provides. In the following we discuss the individual steps.

### Input material

**Figure 1:**
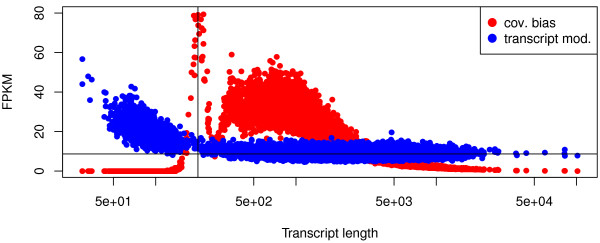
**FPKM bias induced by coverage bias and transcript modifications.** The points represent RefSeq isoforms and the plot shows the length dependency of the FPKM of the sequenced reads for input material where each isoform has the same abundance and where the isoforms exhibit coverage bias or transcript modifications, respectively. The nominal FPKM implied by the isoform abundance and the nominal isoform length is indicated as horizontal line. The fragmentation step in the library preparation generates fragments of an approximate length of 200 nt and causes the sharp peak of highly overestimated FPKM for isoforms having approximately this length.

**Figure 2:**
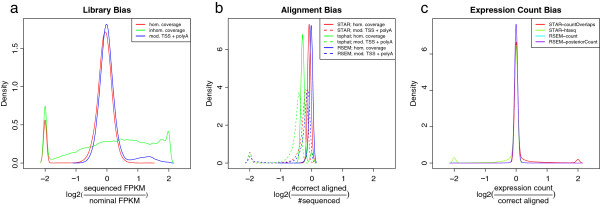
**Biases of the observed FPKM displayed as density distributions.** We visualize the effect of the library **(a)**, alignment **(b)**, and counting biases **(c)** on the observed FPKM. The log2-ratios were constrained to the range [-2, 2] and the density distributions were generated using a band-width of 0.05. The library composition has the biggest impact and causes over- as well as underrepresentation. The aligners do not align all reads correctly but achieve a very high rate for the majority of the genes. If the transcripts exhibit variable transcription start sites and poly-A tails the alignment rate for the short isoforms drops (see the Additional file 1 for the per isoform mapping rates).

How well transcripts are represented in the fragment library to be sequenced may already constrain how accurate genes and isoforms can be quantified in the later steps. The number of reads generated from a given isoform depends on the isoform length, the presence of potential 5′-to-3′ biases, the presence of a poly-A tail and the presence of variations of transcription start relative to the annotated transcription start site. To overcome the dependence of the read counts on the isoform length, FPKM was introduced as expression measure [18]. FPKM builds on the assumption that the number of reads that are generated from an isoform is proportional to the isoform abundance as well as the isoform length. Additionally it normalizes for sequencing depth. A closer analysis (see e.g. [5,12]) shows that this model oversimplifies the situation since the isoform length has to be replaced by an effective length that considers the length distribution of the fragmented RNA. In our simulation we find that the relative abundance of each isoform in the starting material agrees well with the FPKM of the sequenced reads. However, if we allow for coverage bias introduced in the library preparation, then only 54% of all isoforms have an FPKM that is within a factor of two of the nominal value implied by the relative abundance and length of the isoform. Short isoforms are massively overrepresented while long isoforms are underrepresented. A less dramatic deviation occurs if the transcribed sequences are modeled to have variable transcription start sites and a poly-A tail. In this situation, 1343 isoforms are overrepresented by more than a factor of two. The length dependency of the FPKM bias induced by coverage bias and transcript modifications is visualized in Figure 1. The sharp peak of overrepresented isoforms is due to the fragmentation that generates fragments of an approximate length of 200 bp. In Figure 2a, we show the density distribution of the bias factors induced by library properties for the RefSeq genes.

**Figure 3:**
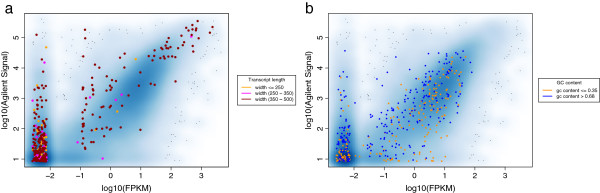
**Length and GC Bias in microarray and RNA-seq data.** The smooth scatterplots compare the expression scores of a single sample measured by RNA-seq and microarray. In 3a we highlight additionally the short isoforms and find that RNA-seq tends to underestimate expression of short isoforms. In 3b we highlight isoforms with extreme GC content. The plot suggests that microarray and sequencing underestimate expression of low GC isoforms, while the high GC isoforms are overestimated by microarray and underestimated by RNA-seq.

We further check in real data for the presence of a transcript length effect and investigate GC bias. We use the microarray and HiSeq RNA-seq (Accession GSE37704) from Trapnell *et al.*[11]. Both datasets have been generated using the same samples. In Figure 3a, we show a smooth scatter plot comparing the microarray signals and the RNA-seq FPKM values. The highlighted short isoforms show that the RNA-seq data underestimates their expression relative to the microarray. If we look at isoforms that are well detected by the microarray (signal intensity > 50) and low abundant in the RNA-seq (FPKM < 0.01) we find that the short isoforms (length < 350 nt) are overrepresented (significance in Fisher’s Exact test is 3.9e-05 with an odds ratio of 3.8). Figure 3b shows the same data but now highlighting isoforms with extreme GC content. Here, the data allows no conclusions since the microarray signals also do have a dependency on GC content. However, the presence of a GC-bias has been shown by Hansen et al. [19] revealing that isoforms with extreme GC content are underrepresented.

The representation biases of the isoforms together with isoform characteristics and more statistics can be found in Additional file 1.

### Alignment

**Figure 4:**
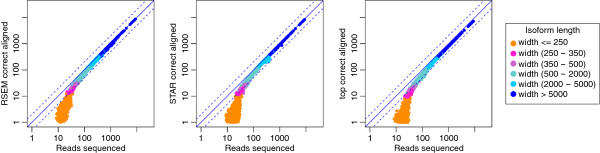
**Isoform length dependence of the alignment rate in the presence of variable transcript start sites and poly-A tails.** If the transcripts are simulated with poly-A tails and variable transcript start sites, the mapping rate of the short isoforms is low. For the visualization at the log-scale we offset the counts by 1 and a small jitter.

We consider an alignment as correct if the alignment coordinates fall within the range of the read-generating isoform. This is sufficient for the read to be counted towards the expression of the isoform. This definition implies that we tolerate if the aligner clips the read ends. We show later that read-end clipping may affect the detection of isoform switching. The medians of the per-isoform alignment rate for different methods and inputs are listed in Table 2. The highest alignment rate (with half the genes having an alignment rate of 99% or more) is achieved if the reads are simulated according to the reference transcripts. Alignment rates drop if the simulation allows for variable read starts and adds poly-A tails. Since short isoforms have proportionally more reads that include the transcript ends, they suffer most from these transcript modifications. Figure 4 shows the length dependency of the alignment rates. The difference in the alignment rates between tophat and STAR roots in the fact that STAR allows more mismatches and supports local alignments. The full table of alignment rates for the individual isoforms is available as Additional file 1. In this comparison we allowed STAR to report up to 30 valid alignments per read. This is different from the default (10). The choice was based on a preliminary study (data not shown) where we found that STAR would produce a low mapping rate for a few large gene families.

**Table 2:** Median value of the per-isoform mapping rate

**Alignment method**	**Hom. coverage**	**Inhom. coverage**	**Mod. TSS + polyA**
RSEM	0.990	0.990	0.898
STAR	0.943	0.943	0.858
tophat	0.814	0.814	0.737

Using the alignment methods implemented by STAR, tophat, and RSEM, and different types of input material, we compute for each isoform the mapping rate and report the median value for all isoforms.

### Abundance estimation

**Figure 5:**
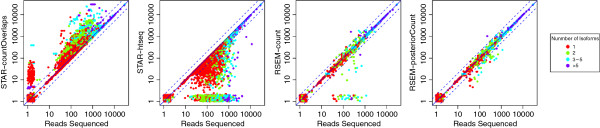
**Comparison of reported expression counts with the number of reads sequenced.** The biases are dominated by the way the reads with multiple alignments are taken into account. Multiple counting leads to overestimation while ignoring multi-reads leads to underestimation. The most accurate results are achieved by the posterior estimates of RSEM.

We first look at gene level expression estimates, which can be derived either by a counting or an isoform abundance approach where the isoform abundances are summed up to yield the gene abundance. As Table 3 shows, the RSEM approach yields a good expression estimate for more than 92% of the genes while the counting approaches only yield an accurate result for 87 to 89% of the genes. The impact of over-/under-counting is visualized in Figure 5. In the case of human genome 3-4% of the genes are strongly affected. Figure 5 also shows that RSEM’s posterior estimation is able to correct some of the reads that are initially incorrectly assigned. For reference we report all gene level scores in Additional file 2.

**Table 3:** Fraction of genes where the expression count bias is less than a factor of two

**Alignment and count method**	**Hom. coverage**	**Inhom. coverage**	**Mod. TSS + polyA**
STAR-countOverlaps	0.892	0.892	0.892
tophat-countOverlaps	0.893	0.893	0.893
STAR-htseq	0.877	0.877	0.877
tophat-htseq	0.883	0.883	0.883
RSEM-count	0.929	0.929	0.929
RSEM-posteriorCount	0.930	0.930	0.930

For each gene we estimate expression in terms of read count and report the fraction of genes for which the estimate is less than a factor of two different from the number of correctly aligned reads. We consider the alignments produced by STAR and tophat in combination with subsequent counting of the overlapping reads using HTSeq and the countOverlaps method. The RSEM software performs alignment and expression counting. Here we list the results for the reported counts as well as the results for the posterior estimates of the counts.

We show the density distributions of the count biases in Figure 2c. The comparison with the library representation and the read alignment biases shows that the counting biases affect the fewest genes.

Additionally, we verify how well RSEM can reproduce the isoform abundances. We find that RSEM does not correctly produce zero counts in the case where a gene has multiple isoforms but only one isoform is expressed. This observation is also reported by Mezlini *et al.*[20]. It is caused by the fact that RSEM tends to bias the expression estimates of the individual isoforms towards the mean value of the corresponding gene locus.

### Differential expression

**Figure 6:**
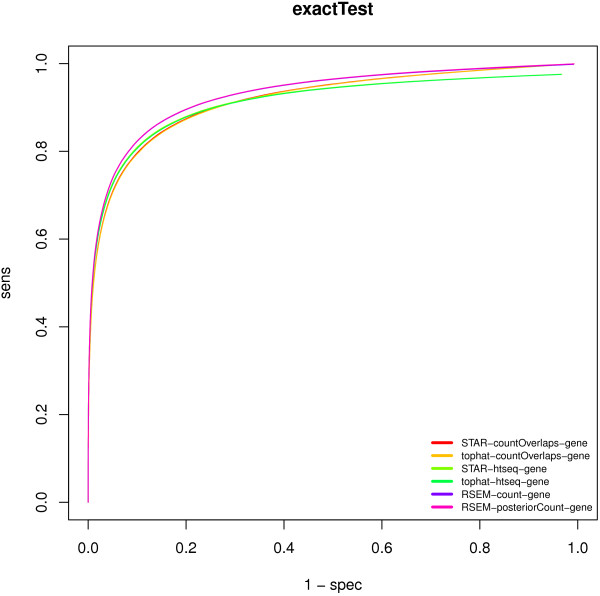
**Receiver-operator-characteristics for the detection of differential expression based on p-values computed with edgeR’s exactTest.** The Area-under-the-curve (AUC) depends on the type of preprocessing. Highest AUC is achieved with RSEM counts reported by RSEM. Other methods score slightly lower with htseq-based counts reaching a plateau that is below 1.0 due to the fact that htseq would not report counts for overlapping gene symbols.

We further generate samples that exhibit differential expression and analyze the ability to assess the differentially expressed genes. To this end, we generate different conditions with three replicates each. The conditions have 10% of the genes upregulated by a factor of two and 10% of the genes downregulated by a factor of two relative to the baseline condition. We simulate biological noise by adding a log-normal noise to the expression fractions of the replicate samples that are used by Flux as input. In total, we simulate 11 different conditions, and test subsequently for differential expression between pairs of conditions. Based on the set of all comparisons, we assess the receiver-operator-characteristic (ROC) for the different test-methods and expression estimates. Table 4 shows the Area-under-the-curve (AUC) values for the exactTest, log-ratio, and t-test applied to different expression estimates. We get AUC above 0.91 when using the exactTest independent of the expression estimate. Interestingly, using the effect-size, i.e. the mean log-ratio as score for differential expression, the AUC performance is even a minute amount higher, again independent of the expression estimate used. Additionally we have included the t-test applied to the log-expression scores. The assumptions of the t-test are not compatible with the count data and the t-test does not make use of the mean-variance relationship that can be found in counting data. We show it here to demonstrate the benefit obtained by using an appropriate count model (as in edgeR). Figure 6 shows the ROC graphs when assessing differential expression using exactTest and using different expression estimates as input. We find that if one uses HTSeq counts as input, the maximum achievable sensitivity is lower than for the other methods. This is due to the fact that for overlapping gene symbols HTSeq does not count the reads and for those genes differential expression cannot be positive. For each combination of counting and testing method, we have computed the per-gene AUC values and report them in Additional file 2. We consider a gene as not detectable by a given method if its AUC value is below 0.1. Table 5 shows that the number of genes not detectable by an individual method ranges from 0 to 506 where the highest number of undetectable genes is observed when using HTSeq counts. In principle, all genes for which reads have been produced, can be detected as differentially expressed if the countOverlaps approach or the RSEM posterior counts are used.

**Table 4:** Area under the curve for the differential expression

**Alignment and count method**	**Exact test**	**Log ratio**	**T-test-log**
STAR-countOverlaps	0.917	0.9191	0.8845
tophat-countOverlaps	0.9162	0.9183	0.8837
STAR-htseq	0.9119	0.916	0.8797
tophat-htseq	0.9109	0.9151	0.8784
RSEM-TPM	0.9264	0.9342	0.8964
RSEM-posteriorTPM	0.9273	0.9355	0.897
RSEM-count	0.9295	0.9328	0.8959
RSEM-posteriorCount	0.9303	0.9338	0.8965

The table shows the overall AUC value for the detection of differential expression. The samples were designed such that each gene was differentially expressed in multiple comparisons. Using different types of input we apply 3 testing scores: exactTest (edgeR package), log-ratio (as an example for effect-size estimation), t-test on the log-expression scores (as a baseline in order to contrast the benefit of using a count-model).

**Table 5:** Number of genes where differential expression was not detectable

**Alignment, count, and test method**	**Number of genes not detectable**
STAR-countOverlaps exactTest	0
tophat-countOverlaps exactTest	0
STAR-htseq exactTest	492
tophat-htseq exactTest	506
RSEM-TPM exactTest	21
RSEM-posteriorTPM exactTest	0
RSEM-count exactTest	19
RSEM-posteriorCount exactTest	0

The comparison demonstrates the power and benefit of the counting model but also shows that if only few replicates--in our case three--are available, the effect-size is a valuable score for differential expression. Corresponding findings were also reported for microarray data [21]. In a related work on comparing hypothesis test based differential expression of counting data, Robles et al. [22] have shown that with 6 replicates there is a remarkable improvement of the power. This power comes mostly from the improved precision on the variance estimate. For RSEM, the distributions of the read counts do not follow strictly a Poisson or negative-binomial model, since the assignment of multi-alignments leads to fractional read counts and the posterior estimation adds additional noise to the estimate. However, we find that the Poisson approximation is reasonable and leads to accurate results.

**Figure 7:**
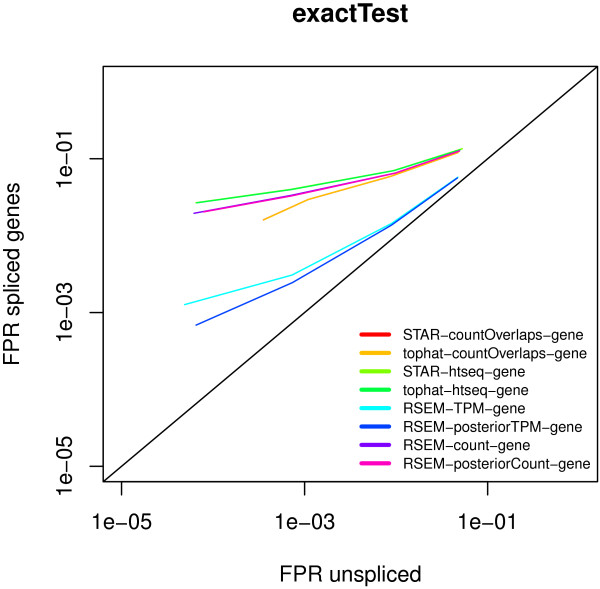
**Isoform switching increases the False Positive Rate (FPR) of differential expression.** If at a gene locus the expressed isoform changes, this leads to an increase in the false positive detection of differential expression based on gene-level counts. For set of significance thresholds we plot the FPRs in the absence and presence of isoform switching. False Positive Detection can only be avoided if expression counts are normalized for isoform length as is the Transcripts-per-Million (TPM) score returned by RSEM.

We additionally evaluate to which extent isoform switching without expression change affects the assessment of the differential expression estimate. We use again conditions with three replicates where only the isoform changes but not the number of transcripts expressed from a given locus. Figure 7 shows that such isoform changes lead to an increased false positive rate (FPR). Isoform switching can lead to significant change in the isoform length and consequently a change in the read count, which is then reported as expression change. The only way to circumvent this is to resort to expression measures that are normalized for isoform length, like FPKM or transcripts per million transcripts sequenced (TPM).

### Isoform switching

**Figure 8:**
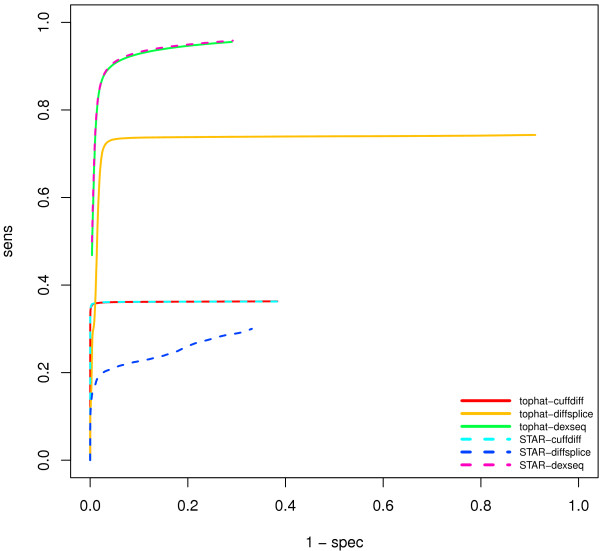
**Receiver-operator-characteristics for the detection if isoform switching.** The performance strongly depends on the alignments and the testing algorithm. In our comparison DEXSeq performs best. DiffSplice only performs well with tophat alignments but not with STAR alignments. The curves do not cover the full range of specificities because algorithms like DEXSeq and cuffdiff only run a test for a given gene locus if the locus satisfies additional constraints on read coverage. If a test was not run, it was considered as no splicing detected.

Finally, we measure the performance of the detection of isoform switching. To this end we use simulated data with three replicates and conditions where either all isoforms of a gene symbol are expressed at the same level or where only one isoform is expressed. We compare the three methods cuffdiff (isoform model based), DEXSeq (based on counts of non-overlapping exon-like segments), and DiffSplice (based on read counts for junctions and exons) in combination with the alignments generated by STAR and cufflinks. We show the ROC curves based on 7347 Genes with multiple isoforms in Figure 8. In Table 6 we report the number of genes for which the per-gene AUC is below 0.1. Overall, the DEXSeq approach has the best performance, followed by DiffSplice and cuffdiff. The remarkable feature is that DEXSeq and cuffdiff work equally well with tophat and STAR alignments, whereas DiffSplice has good performance with tophat but very bad performance with STAR alignments. A closer investigation shows that tophat, despite having less alignments in general, has more intron spanning alignments than STAR. Specifically tophat has 24.1% of the alignments spanning one intron, and 2.3% of the reads spanning two introns, while STAR has 22.7% and 1.8% respectively. This is explained by the fact that STAR can choose a trimmed alignment instead of a full-length spliced alignment, and applies this if only a short fraction of a read reaches into the next exon.

**Table 6:** Number of genes where alternative was not detectable

**Alignment and isoform switching method**	**Number of genes not detectable**
tophat-cuffdiff	4448
tophat-diffsplice	366
tophat-dexseq	109
STAR-cuffdiff	4447
STAR-diffsplice	617
STAR-dexseq	111

The number for cuffdiff is very high because cuffdiff has (with default options) a minimum requirement of 10 reads for a gene locus in order to run the test for isoform switching.

## Conclusions

We have investigated the entire data analysis workflow starting from the input material to the biologically interpretable quantitative results. We find that library content is crucial and biases in the input material do strongly impact subsequent analysis results. To achieve highly accurate mappings for each gene it is essential that the alignment also tolerates variable transcription start sites and poly-A tails. While RSEM models poly-A tails, the aligners tophat and STAR do not. However the latter do cope with variable transcription start sites since they consider transcriptome alignments as well as genome alignments. We further find that overall mapping rates are not sufficiently informative since an aligner may systematically fail to align reads for a given gene. With respect to expression estimation, it is essential to resolve the ambiguous reads. Only by considering the ambiguous alignments an accurate expression estimation is possible for repeated regions or overlapping gene symbols. Differential expression using the count model is powerful but crucially depends on the accuracy of the counts. With as few as 3 replicates the negative binomial test, in our dataset, does not outperform the average effect-size as an indicator of differential expression. Since the read counts that are required as input for the counting tests are not normalized for transcript length they can lead to misinterpretation of isoform switching as differential expression. Detection of isoform switching does not reach the same accuracy as the detection of differential expression. This is due to the high similarity of isoforms and, depending on the gene locus, the potentially small number of aligned reads that do discriminate between the isoforms.

While RNA-seq can give an accurate estimation of gene expression as well as expression differences for the majority of genes, it may miss genes with extreme properties with respect to sequence length, GC content, and homologous regions. The detection of isoform switching may require significantly higher coverage to get satisfactory results. We refer to the additional files for detailed information on how well the transcriptional state of each gene and isoform can be assessed quantitatively.

For future analysis, it will be relevant to consider also single nucleotide polymorphisms in an analysis. Also, it will be interesting to investigate to which extend the results are applicable to other species. The current study assumes that all potential isoforms are known, however in practice the annotated isoforms will not represent the complete transcriptome and additional isoforms that are expressed may have adverse effects and lead to misinterpretations.

## Methods

### Simulated datasets

We used simulated data sets with a baseline expression where each isoform has the same transcript abundance. We consider as transcripts the Refseq transcripts in the hg19 build available from the UCSC Genome Browser web-site. For the differential expression we divide the genes in 10 chunks und choose always 1 chunk for two-fold upregulation and 1 chunk for two-fold downregulation. In total we use 11 conditions with 3 replicates each and each replicate having 10 Mio reads. We create 3 isoform switching datasets from the initial 11 conditions by shifting the entire expression on a gene locus into the first, second and third annotated isoform, respectively. For the methods below, if not mentioned differently, default options were used.

### Read simulation with flux

For simulation we use the Flux read simulator v1.2. We simulate coverage bias by setting the fragmentation substrate to DNA. We choose the default options for the transcript modification to obtain transcripts with variable read starts and poly-A tail. We set both options to NaN and the fragmentation substrate to RNA to produce transcripts that follow exactly the annotated transcripts. We choose as read length 101 bp and use the default error distribution provided by Flux.

### Read alignment

For read alignment, we use tophat v2.0.6 and provided tophat with the RefSeq transcript coordinates as a GTF file so that tophat would also align the reads to the known transcripts. Additionally, we used STAR v2.3.0e with the options“--outSAMstrandField intronMotif --outFilterMatchNmin 20--outFilterMismatchNmax 5 --outFilterMismatchNoverLmax 0.05--outFilterMultimapNmax 30″ that add additional strand information for spliced alignments, limit the number of mismatches to 5 and that increase the number multimapping for reads to 30. We use RSEM v1.2.3 for read alignment to the transcriptome and use RSEM also to compute expression estimates.

### Expression estimation

We compute expression with HTSeq v0.5.3p9 using the script HTS.scripts.count, as well as by using the countOverlaps method in the Bioconductor package GenomicRanges v1.10.7 and the expression output generated by RSEM.

### Differential expression

We compute differential expression with the exactTest method in the Bioconductor package edgeR v3.0.6, and the t-test in the package genefilter v1.40.0. Additionally we compute the log-ratio as an effect size. We did not apply any normalization with respect to sequencing depth, since all samples had the same sequencing depth.

### Isoform switching

For the detection of isoform switching we use the Bioconductor package DEXSeq v1.4.0 which also provides a python scripts to generate the read counts for the pseudo-exons that are needed as input for the hypothesis test. The other two methods are cufflinks v2.1.1 (with the option for multi-read correction) and DiffSplice v0.1.1. We used all genes that were commonly detectable by DEXSeq and cuffdiff. This excludes genes where the same gene symbol is associated with more than one genomic locus because DEXSeq can not handle such situations. These genes have been removed and the GTF File from UCSC has been preprocessed with DEXSeq’s python script dexseq_prepare_annotation.py. DiffSplice does not make use of the gene annotation at all but reports alternatively spliced modules (ASM) independent of the annotation. In order to get comparable results we matched the ASM to the overlapping gene symbols.

## Competing interests

The authors declare that they have no competing interests.

## Authors' contributions

HR conceived the study, generated the simulation data, performed the data analysis, and wrote the manuscript. LO contributed to the data analysis and the manuscript. GT and LS wrote the elements of the data analysis pipeline and evaluated the performance. RS provided input for the manuscript and critically reviewed it. All authors read and approved the final manuscript.

## Supplementary Material

Additional file 1 **Summary table that holds the statistics per isoform.** Detailed numeric results for each transcript ID (i.e. isoform) with respect to bias factors and mapping rates. Click here for file

Additional file 2 **Summary table that holds the statistics per gene symbol.** Detailed numeric results for each gene symbol with respect to reads sequenced, bias factors, mapping rates, and Area-under-the-curve (AUC) values. Click here for file
